# *Trypanosoma cruzi* extracellular amastigotes and host cell signaling: more pieces to the puzzle

**DOI:** 10.3389/fimmu.2012.00363

**Published:** 2012-11-30

**Authors:** Éden R. Ferreira, Alexis Bonfim-Melo, Renato A. Mortara, Diana Bahia

**Affiliations:** ^1^Disciplina de Parasitologia, Departamento de Microbiologia, Imunologia e Parasitologia, Escola Paulista de Medicina, Universidade Federal de São PauloSão Paulo, SP, Brazil; ^2^Departamento de Biologia Geral, Instituto de Ciências Biológicas, Universidade Federal de Minas GeraisBelo Horizonte, MG, Brazil

**Keywords:** extracellular amastigotes, cell invasion, signaling, mevalonate kinase, protein kinase D

## Abstract

Among the different infective stages that *Trypanosoma cruzi* employs to invade cells, extracellular amastigotes (EAs) have recently gained attention by our group. This is true primarily because these amastigotes are able to infect cultured cells and animals, establishing a sustainable infective cycle. EAs are thus an excellent means of adaptation and survival for *T. cruzi*, whose different infective stages each utilize unique mechanisms for attachment and penetration. Here we discuss some features of host cell invasion by EAs and the associated host cell signaling events that occur as part of the process.

## INTRODUCTION

The parasite *Trypanosoma cruzi* employs a variety of distinct strategies to facilitate invasion of mammalian cells. For instance, multiple infective stages [e.g., bloodstream trypomastigotes, metacyclic trypomastigotes, and extracellular amastigotes (EAs)], varying strains and isolates, as well as differing infectivities have been widely acknowledged as significant obstacles to effective treatment. The multitude of invasive strategies employed by *T. cruzi *represents an important survival advantage for the organism and allows for the remarkably wide range of mammalian hosts affected by this parasite. More specifically, *T. cruzi *infects over 100 species from several orders and develops *in vivo* within a variety of cellular niches, including macrophages, muscle tissue, epithelial cells, fibroblasts, and nerve cells. The ability of *T. cruzi* to invade, persist and adapt in both invertebrate and vertebrate hosts is multifactorial, and depends on both the parasite and host fitness. The cellular communication between parasite and its host is constant and has evolved to be relatively benign, as killing the host would not be advantageous to the parasite.

Extracellular amastigotes are either prematurely released from infected cells or generated by the extracellular differentiation of released trypomastigotes (reviewed in Lima et al., 2010). During the acute phase of *T. cruzi* infection within mice, EAs represent 10% of circulating parasite forms and are capable of sustaining an infective cycle in the mammalian host and cells (Andrews et al., 1987).

**FIGURE 1:**
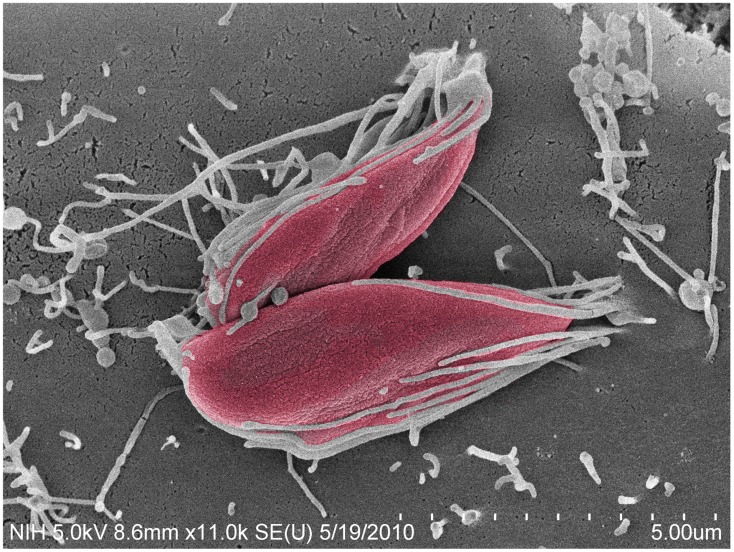
***Trypanosoma cruzi* G strain amastigotes interact with microvilli on the surface of HeLa cells.** Parasites (colored in red) imaged by field emission-scanning electron microscopy, attach to HeLa cell surface microvilli. Magnification bar = 5 μm.

Among the EA stage, *T. cruzi* I strains (such as the G and Tulahuén strains) are more infective than *T. cruzi* II and VI strains (such as the Y and CL strains; Fernandes and Mortara, 2004; Mortara et al., 2005). In contrast to trypomastigotes, the recruitment of actin is central to the uptake of EA forms in mammalian host cells (**Figure 1**), including HeLa cells, the model cell type used in our studies (Mortara, 1991; Procópio et al., 1998; Mortara et al., 2005).

Here we aim to highlight aspects of host cell invasion by EAs and introduce novel findings including the involvement of molecules from both host cell and the parasite that trigger host cell signaling events.

## MAMMALIAN CELL INVASION BY EXTRACELLULAR AMASTIGOTES: THE STORY BEGINS

Prior to the mid 1980s, chronic infection with *T. cruzi* was assumed to be sustained by few trypomastigotes in the bloodstream that had escaped the immune response and invaded new cells. The fate of amastigotes at that time was thought to be restricted to the intracellular growth of the parasite. However, pioneering studies (Behbehani, 1973; Nogueira and Cohn, 1976; Lanar, 1979; Abrahamsohn et al., 1983; McCabe et al., 1984; Carvalho and de Souza, 1986) demonstrated that amastigotes shared some physiological characteristics with trypomastigotes, such as the ability to invade and develop within cells *in vitro* and the ability to infect mice. For instance, McCabe et al. (1984) demonstrated that amastigotes isolated from the spleen of mice infected with three different strains of *T. cruzi* (Y, MR, and Tulahuén) were able to infect, survive, and replicate within professional and non-professional (L929 cell line) phagocytes. In a different study, Carvalho and de Souza (1986) obtained highly purified amastigotes (Y strain) from the macrophage-like cell line J774G8. These amastigotes were effectively ingested by macrophages and were able to initiate intracellular replication. Parasite development* in vitro* was measured by means of light microscopy. Moreover, purified amastigotes were highly infective when inoculated into mice, generating high parasitemia and even death (McCabe et al., 1984; Carvalho and de Souza, 1986).

Andrews et al. (1987) found that amastigotes accounted for 10% of circulating forms in the blood of mice during the acute phase of infection. Furthermore, this amastigote form was shown to express a specific surface glycoprotein that was designated Ssp-4. These observations were corroborated when Ley et al. (1988), by using transmission and scanning electron microscopy, demonstrated that extracellular axenically derived amastigotes (i.e., trypomastigote–amastigote differentiation in axenic medium) were able to multiple within cells *in vitro.* When injected intraperitoneally, the amastigote forms were as infective to mice as trypomastigotes. Researchers were also able to measure the infectivity and the multiplication rates of the parasites inside cultured cells (Ley et al., 1988). The process of trypomastigote–amastigote differentiation in axenic medium can be accelerated by lowering the pH of the medium (Tomlinson et al., 1995). EAs can also be obtained from axenic culture media specifically designed for isolating amastigotes (Pan, 1978; Rondinelli et al., 1988). Extracellular (axenically derived) amastigotes resembled intracellular amastigotes with regard to cell body and kinetoplast morphology, and cell surface expression of Ssp-4 (Ley et al., 1988; Barros et al., 1997; Silva et al., 1998).

*In vitro*, amastigotes are able to infect and complete their life cycle within phagocytic and non-phagocytic host cells, possibly using distinct molecular mechanisms for invasion and escape from the parasitophorous vacuole (Pan, 1978; Carvalho and de Souza, 1986; Ley et al., 1988; Mortara, 1991; Procópio et al., 1998, 1999; Andreoli and Mortara, 2003; Mortara et al., 2005; Florencio-Martínez et al., 2010; Bambino-Medeiros et al., 2011).

*In vivo*, EAs were intraperitoneally inoculated into groups of five A/J mice and all mice were not only infected, but died after 18–25 days. Two trypomastigote parasitemia peaks were observed on days 7 and 12. Significantly higher numbers of parasites were observed during the second peak in mice that were inoculated with Y strain amastigotes (Ley et al., 1988). EAs were also resistant to complement-mediated lysis (Carvalho and de Souza, 1986; Iida et al., 1989; Fernandes and Mortara, 2004), a feature thought to be required for infectivity. The general conclusion from these studies was that EAs, once prematurely released from dead cells, may persistently infect resident macrophages by initiating an alternative subcycle and/or using an alternative pathway of invasion. It was hypothesized that these events could be crucial for the maintenance of tissue infection and inflammation (Scharfstein and Morrot, 1999) within the mammalian host.

In 1991, the mechanisms by which amastigotes invade non-phagocytic cells began to be determined. Mortara (1991) observed that amastigotes interact with microvilli on the dorsal surface of HeLa cells leading to microvillus aggregation. This event can be followed by microfilament clustering observable by phalloidin staining and fluorescent microscopy. By a combination of fluorescence and scanning electron microscopy, it was observed that actin aggregates underneath the sites of amastigote adhesion and forms a small clump (Mortara, 1991) that was later called the actin cup-like structure (Procópio et al., 1999). Interestingly, cytochalasin D did not significantly affect parasite attachment but the disruption of cellular microfilaments greatly inhibited amastigote entry (Mortara, 1991; Procópio et al., 1998). In 1998, the actin cup-like structures beneath the amastigotes at HeLa cell entry sites were assessed by confocal microscopy (Procópio et al., 1998). In that study, gelsolin, an actin-binding protein was overexpressed in NIH 3T3 fibroblasts. This resulted in a large increase in the internalization of amastigote forms resulting from enhanced microfilament rearrangement. Components of the HeLa cytoskeleton, integrins, and extracellular matrix, such as α-actinin, ABP_280_, gelsolin, α5β5 integrin, laminin, and fibronectin, accumulated along with actin at the sites of EA entry (Procópio et al., 1999).

## EAs MOLECULES OF ADHESION AND SECRETION: INVASION AND SIGNALING

Adhesion is a crucial initial step for microorganisms to invade any cell. Association of amastigotes with host macrophages was shown to be mediated by the macrophage mannose receptor (MR) and mannose-binding protein (MBP; Kahn et al., 1995). MR and MBP are C-type lectins. MBP strongly and stably binds to amastigotes, and this interaction may contribute to parasite opsonization and cellular invasion. MBP–amastigote interaction is stage specific, can be inhibited by mannan, and requires calcium (Kahn et al., 1996). The amastigote ligands for MBP include SA85-1 and other related surface glycoproteins (Kahn et al., 1996). IFN-γ down-regulates MR expression and thus inhibits parasite invasion and replication within macrophages. It has recently been reported that *in vivo *infection by G strain (TcI) EAs is inhibited by IFN-γ production (Rodrigues et al., 2012). Fibronectin also appears to bridge these initial binding steps (Tulahuén strain; Noisin and Villalta, 1989; Procópio et al., 1999). Our group has previously shown that carbohydrate epitopes expressed on the surface of EAs may also play a role in parasite adhesion to HeLa cells since monoclonal antibodies inhibited the process (Silva et al., 2006).

Cruz et al. (2012) demonstrated that the treatment of HeLa cells with recombinant amastin, a surface glycoprotein abundant in amastigotes, reduced the infectivity of EA forms. Conversely, the ectopic *T. cruzi* expression of amastin accelerated differentiation of amastigotes into trypomastigotes. These results positioned amastin as a potential amastigote molecule able to modulate EA invasion and differentiation within mammalian cells. In addition, amastin might participate in specific host cell signaling culminating in EA invasion.

*Trypanosoma cruzi *EAs may also secrete proteins in order to aid their attachment and entry into HeLa cells. Silva et al. (2009) identified a hypothetical protein of 21 kDa with no known orthologous in other species. So-called P21 is secreted by the parasites and enhances amastigote invasion, especially when the recombinant protein is added to host cells together with parasites as part of HeLa cell invasion assays. Researchers concluded that P21 triggers unknown host cell signaling events that lead to parasite internalization.

Moonlighting enzymes or protein moonlighting refers to a phenomenon in which a protein can perform more than one function, including unexpected functions (Jeffery, 2009). Many proteins that moonlight are enzymes involved in glycolysis, an ancient universal metabolic pathway. It has been suggested that as many as 7 of 10 proteins in glycolysis and 7 of 8 enzymes of the tricarboxylic acid cycle exhibit moonlighting behavior. Our group has recently characterized a moonlighting protein in *T. cruzi* called mevalonate kinase (MVK), an enzyme involved in isoprenoid synthesis. As part of the *T. cruzi *infection process, this moonlighting protein is involved both in parasite invasion and host cell signaling (Ferreira et al., unpublished). There are numerous intermediates in the mevalonate biosynthetic pathway that play important roles in the post-translational modification of a multitude of proteins involved in intracellular signaling. These intermediates in the mevalonate biosynthetic pathway are essential regulators of cell growth/differentiation, gene expression, protein glycosylation, and cytoskeletal assembly.

Microarray analysis of EA mRNAs has demonstrated that MVK displays higher expression within G (more infective) than in CL (less infective) parasites, suggesting an important role of the MVK pathway in EA infectivity (Ferreira et al., unpublished). We have obtained data indicating that MVK may be a modulator in EA invasion and could become an important target in the development of new drugs to treat Chagas’ disease. Moreover, in *T. cruzi*, MVK is secreted and likely participates in the modulation of HeLa cell signaling leading to EA cell invasion. These observations reveal new possibilities for the study of moonlighting protein evolution and function within intracellular parasites.

## EAs AND HOST CELL SIGNALING

The first experiments to describe signaling events induced by EAs in non-phagocytic cells were performed in 1998. Procópio et al., (1998) used a protein kinase C inhibitor, staurosporine, and also a tyrosine kinase inhibitor, genistein, to treat HeLa cells before invasion assays with EA. Curiously, staurosporine had no effect whereas genistein moderately inhibited the invasion of EAs in HeLa cells. In Vero cells, genistein had no effect but staurosporine inhibited EA invasion by 82%. The role of RhoA GTPases in host cell invasion by EAs of both G and CL strains of *T. cruzi* was subsequently studied. Rho GTPases regulate three separate signal transduction pathways, linking plasma membrane receptors to the assembly of distinct actin filament structures. Fernandes and Mortara (2004) used non-polarized MDCK cells transfected with different Rho GTPase constructs (RhoA, Rac1, and Cdc42). EA invasion was particularly high in MDCK cells overexpressing either wild type or constitutively active Rac1. Consistently, EA invasion was specifically reduced in the corresponding dominant negative line, suggesting a key role for Rac1-GTPase in the invasion process (Fernandes and Mortara, 2004). On the other hand, in contrast to a number of bacterial invasion mechanisms (Mounier et al., 2003), amastigote invasion seems to be independent of Cdc42.

Sonicated extracts from G or CL strain of EA induced transient enhancement in HeLa cell intracellular Ca^2^^+^ levels in a dose-dependent manner. Inhibition of HeLa cell intracellular Ca^2^^+^ mobilization by thapsigargin or caffeine moderately reduced the infectivity of both G and CL strains (Fernandes et al., 2006). Thus, EAs of both strains trigger calcium signaling in HeLa cells that may be important for the success of EA invasion. Adenylyl cyclase based activation of HeLa cells by exposure to forskolin did not affect infection by either strain. The activation of PI3 kinase in host cells appears to be required for invasion by either the G or CL strain since treatment of HeLa cells with wortmannin reduced EA infectivity (Fernandes et al., 2006). It has also been described that the use of PI3 kinase inhibitors impairs EA (Y strain) internalization into peritoneal macrophages by 60% (Barrias et al., 2010).

We have recently observed that the treatment of non-phagocytic cells with a Src-family tyrosine kinase (SFK) inhibitor, in the absence of serum, reduces EA (G strain) invasion (Bahia et al., unpublished). In addition, EA of the G strain induced time-dependent HeLa cell phosphorylation of SFKs, whose members include Src, Lyn, Fyn, Lck, Yes, and Hck. At this stage, however, the precise Src members involved in this pathway have not been identified. These results suggest that EAs may also exploit the host cell Src pathway in order to invade cells.

## EAs AND THE HOST CELL POINT OF VIEW

Host cell plasma membrane microdomains were also shown to be involved in EA *T. cruzi *entry into non-phagocytic cells. Membrane microdomains, also known as lipid rafts, play a unique role in signal transduction by compartmentalizing cell receptors and subsequently boosting downstream signaling to the effectors molecules (Simons and Ikonen, 1997). Cholesterol, the major component of membrane lipid rafts of Vero or HeLa cells was disrupted by methyl β-cyclodextrin (MβCD) and then infected with EAs of G (TcI) and CL (TcVI) strains (Fernandes et al., 2007). Removal of cholesterol from both host cell lines significantly decreased the invasion index of EAs, indicating that host cell cholesterol and/or membrane organization is important for the entry process of both infective forms. After cholesterol repletion, the invasion index of G strain EAs was almost fully recovered. Subunit B of cholera toxin binds to the GM1 ganglioside, a marker for lipid rafts, and the treatment of non-phagocytic cells with CTB (cholera Toxin-B) also decreased EA invasion. In macrophages, the participation of lipid rafts in the internalization of EAs has also been described (Barrias et al., 2007).

Barrias et al. (2010) showed that dynamin, a large GTPase that belongs to a protein superfamily with a critical role in endocytic membrane fission events, plays a role in EA internalization into peritoneal macrophages. Researchers treated macrophages with dynasore, a reagent that has the ability to block the GTPase activity of dynamin, and observed a marked inhibition in the internalization of EAs. However, dynasore did not significantly interfere with parasite adhesion to host cells.

Bambino-Medeiros et al. (2011) showed that amastigote invasion also involves host cell surface heparan sulfate proteoglycans, which localize to signaling membrane microdomains such as lipid rafts. By treating EAs of the Dm28c strain with heparin or heparan sulfate, researchers observed inhibition of EA entry into primary cultures of cardiomyocytes. The authors hypothesized that the binding of amastigote heparin-binding protein could activate different signaling pathways, such as phosphorylation of cortactin by Src activation that would lead to actin polymerization and amastigote entry.

**FIGURE 2:**
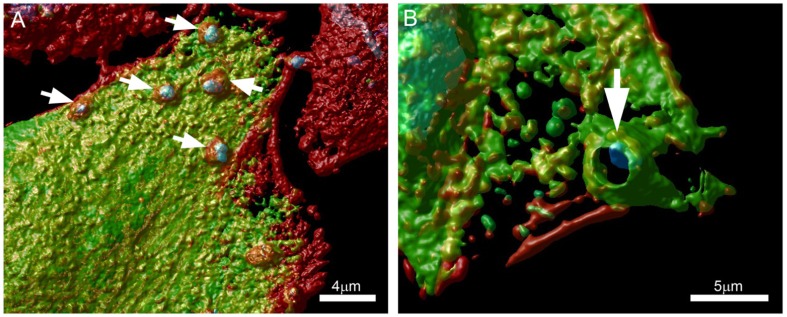
**Extracellular amastigotes (EAs) were incubated for 1 h with (A) Vero or (B) CHO cells transfected with a GFP tagged PKD (green) and stained with phalloidin-TRITC (actin) and DAPI (cells nuclei)**. Arrows show EA recruiting PKD at the invasion sites. Confocal microscopy followed by Huygens Surface Rendering (www.svi.nl). Magnification bars = 4 μm **(A)** and 5 μm **(B)**.

**FIGURE 3:**
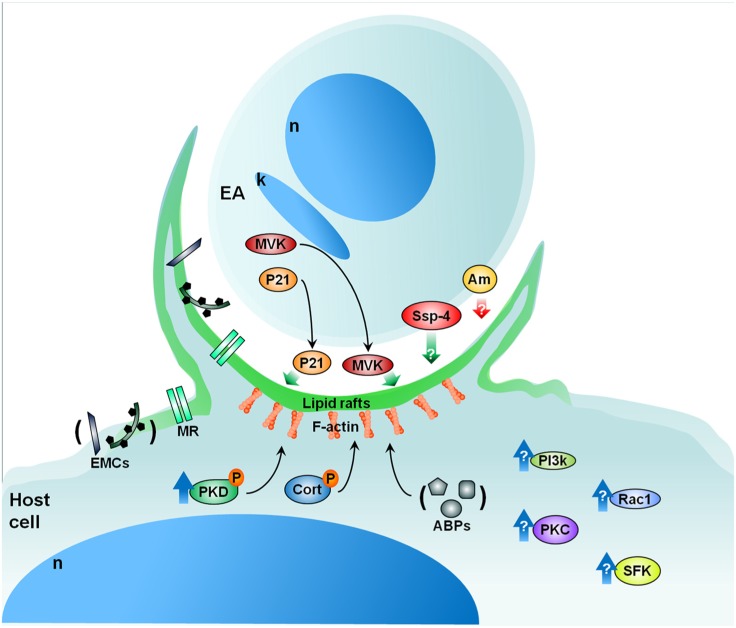
**Proposed model for EA signaling**. EAs secrete factors such as P21 and MVK that aid in EA uptake by cells in culture. EAs also present specific glycoproteins (i.e., Ssp-4) important for attachment and penetration while other EA glycoproteins (i.e., amastins-Am) may negatively modulate amastigote invasion. From the cellular perspective, lipid rafts are essential to parasite internalization in both macrophages and epithelial cells. Furthermore, the mannose receptor of certain cells may recognize mannose residues expressed by EAs which also participate in EA uptake. Upon contact, EAs recruit PKD, actin (F-actin), and other actin-binding proteins (ABPs) such as cortactin (Cort), gelsolin, and α-actinin. Additionally, EAs likely activate Src-family kinase (SFK), PKC, and Rac1 signaling in host cells. The phosphatidylinositol 3-kinases (PI3k) pathway may also be involved in EA internalization. Cellular heparan sulfate and fibronectin are also important in the process of EA internalization (extracellular matrix components – ECMs). Red and green arrows: negative and positive modulation of the invasion, respectively; blue arrows: host protein activation; black arrows: recruitment. P, phosphorylation; n, nucleus; k, kinetoplast; MVK, mevalonate kinase; MR, mannose receptor. See text for more details.

Along these lines, we are currently evaluating the role of protein kinase D (PKD) and cortactin in EA uptake by HeLa cells (Bonfim-Melo, unpublished). PKD is a family of multidomain enzymes (PKD1, 2, and 3). PKD lies downstream of PKCs in a novel signal transduction pathway implicated in the regulation of multiple fundamental biological processes. At the leading edge of migrating cells, active PKD co-localizes with F-actin, Arp2/3, and cortactin. Cortactin has emerged as a key signaling protein in cellular processes such as endocytosis and tumor invasion by interacting with and/or altering the cortical actin network. PKD is an upstream regulator of cortactin. EAs not only recruit PKD (**Figure 2**) and cortactin to the invasion sites of epithelial non-phagocytic cells, but also induce the phosphorylation of these proteins (Bonfim-Melo, unpublished). These results suggest that unexpected novel roads may also be utilized by *T. cruzi* to invade cells. In order to summarize, the putative cellular molecules and signaling pathways used by EAs are presented in **Figure 3**.

## CONCLUDING REMARKS

Extracellular amastigotes of *T. cruzi* are an infective parasite stage that mobilizes molecules and pathways distinct from those engaged by the classical infective trypomastigote forms. This repertoire of molecules may include not only previously characterized carbohydrate epitopes and the P21 secreted component, but also novel components described here such as the secreted form of MVK and the cortactin–PKD pathway.

## Conflict of Interest Statement

The authors declare that the research was conducted in the absence of any commercial or financial relationships that could be construed as a potential conflict of interest.
